# Lower Specificity of the European Society of Cardiology 2023 Diagnostic Criteria for Infective Endocarditis When Spondylodiscitis Is Regarded as a Vascular Phenomenon

**DOI:** 10.1093/cid/ciae223

**Published:** 2024-04-24

**Authors:** Torgny Sunnerhagen, Magnus Rasmussen

**Affiliations:** Division of Infection Medicine, Department of Clinical Sciences Lund, Lund University, Lund, Sweden; Clinical Microbiology and Infection Control, Region Skåne Office for Medical Services, Lund, Sweden; Division of Infection Medicine, Department of Clinical Sciences Lund, Lund University, Lund, Sweden; Department of Infectious Diseases, Skåne University Hospital Lund, Sweden

**Keywords:** infective endocarditis, spondylodiscitis, bacteremia, *Staphylococcus aureus*, diagnostic criteria

## Abstract

The ESC diagnostic criteria for infective endocarditis (IE) added spondylodiscitis as a minor criterion. This resulted in that 11 of 1807 patients with *Staphylococcus aureus*, streptococcal, or *Enterococcus faecalis* bacteremia, were reclassified from possible to definite IE, of whom only two were treated as IE.

Infective endocarditis (IE) and spondylodiscitis (SD) are 2 types of infection that result from a hematogenous spread of bacteria to the heart valves or the spine, respectively. These conditions sometimes occur simultaneously, but only a minority of patients with IE have SD [1] and only a minority of patients with SD have IE [2]. The typical feature of IE is the formation of vegetations on the heart valves that can detach and lead to embolization in distant organs. When IE and SD is present simultaneously, either condition can occur first. SD is therefore not necessarily a result of embolization from the IE vegetation. The European Society for Cardiology (ESC) recently published diagnostic criteria for IE presented in the ESC2023 guidelines for the management of endocarditis. These criteria state that SD should be regarded as a vascular phenomenon, that is, an embolization, and thus constitute a minor criterion for the diagnosis of IE [3]. Previous versions of the Duke criteria as well as the Duke–International Society for Cardiovascular Infectious Diseases (ISCVID) criteria, also presented in 2023, do not regard SD as a vascular phenomenon [4, 5], and the ESC2023 criteria provide no explanation for addition of SD as a minor vascular criterion.

We investigated how the addition of SD as a minor vascular criterion affects the performance of the ESC2023 diagnostic criteria in relation to the Duke–ISCVID criteria in a cohort of patients with bacteremia with typical IE-causing pathogens. These cohorts have previously been used to develop risk stratification systems for IE [6–8]. In total, 1807 episodes of bacteremia were reanalyzed: 1098 with *Staphylococcus aureus* [8], 312 with non–beta-hemolytic streptococci (generation cohort) [7], and 397 with *Enterococcus faecalis* (generation cohort) [6]. The original reports used the ESC2015 definition of IE, which led to 132 episodes (7.3% of episodes) being classified as definite IE. Sixty-eight IE episodes were identified in *S. aureus* bacteremia (6.2%), 20 in streptococcal bacteremia (6.4%), and 44 in *E. faecalis* bacteremia (11%). Using the Duke–ISCVID criteria, an additional 2 episodes were reclassified to definite IE, 1 of which was perceived and treated as IE [9].

In our cohort, SD was identified in 97 episodes (8.8%) of *S. aureus* bacteremia, 8 episodes (2.6%) of streptococcal bacteremia, and 9 episodes (2.3%) of *E. faecalis* bacteremia. Using the ESC2023 criteria, recognizing SD as a minor vascular criterion, a new minor criterion was identified in 87 (*S. aureus*), 7 (streptococci), and 9 (*E. faecalis*) episodes, respectively, since some patients had another vascular phenomenon. This resulted in an additional 11 episodes being reclassified from possible IE (with Duke–ISCVID) to definite IE (with ESC2023). Of the reclassified episodes, 6 were caused by *S. aureus*, 1 by streptococci, and 4 by *E. faecalis.* A description of the episodes is given in Table 1. In 9 of these 11 episodes, the patients were not perceived to have and were not treated for IE. None of the patients had findings suggestive of IE on echocardiography or other imaging modalities. None of the patients experienced a relapse within 6 months. If the decision to treat a patient as having IE was used as the reference standard, the specificity decreased by 9 episodes, corresponding to a decrease in specificity from 100% with the Duke–ISCVID criteria to 99% with the ESC2023 criteria. The sensitivity of the ESC2023 criteria was increased by 2 episodes in our cohorts, using the decision to treat as IE as the standard, corresponding to an increase in sensitivity from 80% with the Duke–ISCVID to 81% with ESC2023.

**Table 1.** Features of Patients Reclassified to Definite Infective Endocarditis (IE) Using the European Society of Cardiology 2023 Criteria for Diagnosis of IE

Age, Sex	Bacteria	Treated as Infective Endocarditis	Number of Positive Blood Cultures	Echocardiography	Positron Emission Tomography–Computed Tomography	Minor Criteria	Acquisition	Comments
40, Female	*Staphylococcus aureus*	No	2	TTE-neg	ND	Fever, SD, PWID	Community	Also arthritis
51, Male	*S. aureus*	No	2	TEE-neg	ND	Fever, SD, PWID	Community	Only 6 d IV antibiotics
55, Male	*S. aureus*	No	2	TTE-neg	ND	Fever, SD, PWID and pIE	Community	…
58, Male	*S. aureus* (methicillin-resistant *S. aureus*)	No	2	TEE-neg	ND	Fever, SD, PWID	Community	…
29, Male	*S. aureus*	Yes	2	TEE-neg	ND	Fever, SD, PWID	Community	Also arthritis
50, Male	*S. aureus*	Yes	2	TEE-neg	ND	Fever, SD, PWID	Community	Arthritis and empyema
78, Female	*Streptococcus gordonii*	No	2	TTE-neg	ND	Fever, SD, NVD	Community	…
77, Male	*Enterococcus faecalis*	No	2	TEE-neg	ND	Fever, SD, NVD	Community	Only 5 d IV antibiotics
63, Male	*E. faecalis*	No	2	TEE-neg	ND	Fever, SD, PV, pIE	Community	…
80, Male	*E. faecalis*	No	3	TEE-neg	Neg	Fever, SD, PV	Nosocomial	Positron emission tomography–computed tomography-neg for IE but shows SD
74, Female	*E. faecalis*	No	2	TTE-neg	ND	Fever, SD, CIED	Community	**…**

Abbreviations: CIED, cardiac implantable electronic device; IE, infective endocarditis; IV, intravenous; ND, not done; neg, negative; NVD, native valve disease; pIE, previous infective endocarditis, PV, prosthetic valve; PWID, person who injects drugs; TEE, transesophageal echocardiography; TTE, transthoracic echocardiography; SD, spondylodiscitis.

SD is most often seen without IE. This demonstrates that SD is not commonly caused by embolization from an IE. The hematogenous spread of bacteria to the spine should therefore not be regarded as a vascular phenomenon as suggested in the ESC2023 criteria. Moreover, regarding SD as a minor criterion, at least in our bacteremia cohort, decreases specificity of the criteria with only a small increase in sensitivity. Interestingly, all 6 episodes of *S. aureus* bacteremia that were reclassified to definite IE were found in persons who inject drugs. Two of these episodes were treated as IE, whereas 4 were not. Four episodes of *E. faecalis* bacteremia were reclassified from possible to definite IE, and in proportion to the total number of episodes, reclassification was more common in *E. faecalis* than in *S. aureus* bacteremia. These 4 episodes were in persons with different types of predisposition for IE, and interestingly, none of them was treated for IE. There was no relapse in bacteremia in episodes that did not receive IE treatment. However, it should be kept in mind that a long per oral antibiotic treatment was given in all episodes, as this is the standard treatment of SD. This can possibly lead to cure of a missed IE.

In another recent study, also on bacteremia with *S. aureus*, the authors found that the addition of SD as a vascular phenomenon appeared to be unhelpful for the performance of the ESC2023 criteria [10]. Based on the above arguments, we suggest that the ESC2023 guidelines remove SD from the list of vascular phenomena/minor criteria. Moreover, we propose that the endocarditis community agree on 1 set of diagnostic criteria for IE by harmonizing the Duke–ISCVID and ESC2023 criteria. This would facilitate both care for patients with IE and research on IE.

## Notes

***Acknowledgments.*** The authors thank all of the coworkers who participated in the original data collection.

***Data availability statement.*** Pseudonymized data will be shared upon reasonable request.
